# Root anatomy and canal morphology of mandibular first premolars in a Chinese population

**DOI:** 10.1038/s41598-017-00871-9

**Published:** 2017-04-07

**Authors:** Lei Dou, Duojiao Li, Tingting Xu, Yin Tang, Deqin Yang

**Affiliations:** 1 Department of Conservative dentistry and Endodontics, Stomatological hospital of Chongqing medical university, Chongqing, China; 2 Chongqing key Laboratory for Oral Diseases and Biomedical Sciences, Chongqing, China; 3 Chongqing Municipal Key Laboratory of Oral Biomedical Engineering of Higher Education, Chongqing, China; 4 Zunyi Medical College, Zunyi, Guizhou China; 5 University of Southern California Herman Ostrow School of Dentistry, Los Angeles, CA USA

## Abstract

This study was to investigate root anatomy and root canal morphology of mandibular first premolars in a Chinese population. 178 human permanent mandibular first premolars extracted from a native Chinese population were collected, scanned using micro-computed tomography and reconstructed three-dimensionally. The number of roots and canals, canal configuration and radicular grooves were investigated. The root canal morphology was categorized according to Vertucci’s criteria. The radicular grooves were scored according to the Arizona State University dental anthropology scoring system (ASUDAS), and the correlation between scores for radicular grooves and root canal morphology was analyzed. Almost all the samples were single-rooted (99.4%). 64.04% of teeth possessed type I canal systems, whilst 34.27% had two canals and 1.69% had three canals. According to ASUDAS, the scores of radicualr grooves were 56.74%, 16.85%, 12.36%, 10.11%, 3.37% and 0.56% respectively from grade 0 to grade 5. The roots with radicular grooves (grade 3 or 4) were defined as Tome’s anomalous root and these roots have a high incidence of C-shape configurations (66.67%) and multiple-canal systems (100%). There is complicated variation of the root anatomy and canal morphology of mandibular first premolars in southwestern Chinese population, which needs special attention and careful assessment for endodontic treatment.

## Introduction

A comprehensive knowledge of external and internal anatomy of human teeth is essential for dental procedures. The human mandibular first premolars are well known for their difficulties in endodontic technique as it pose great challenge for endodontic treatment as a result of the variations in its root canal morphology^1^. An appreciable range of variations with relatively high incidence of abnormalities have been reported for this type of teeth^2^. Furthermore the variations in the root canal morphology have been closely linked to nonsurgical root canal treatment failures and high frequency of endodontic flare-ups^3^.

Radicular grooves or depressions on the external root surfaces are often associated with the ovoid-shaped or other complex configuration in the cross section. Tome’s root is one of those external roots anatomy variations in human permanent mandibular first premolars reported by previous study^4^, which refers to the radicular or developmental grooves of different levels that are considered to be associated with the evolution from single root to multiple roots in mandibular first premolars. Tome’s root can be graded from 0 to 5 according to the Arizona State University dental anthropology scoring system (ASUDAS)^4^. Tome’s anomalous roots equivalent to grades 3 to 4 of ASUDAS classification.

Root anatomy and root canal morphology usually vary in different ethnic groups^5^. An in depth study of root canal configuration with emphasis on different races or region is necessary. Although several studies on root canal and morphology have been carried out in some regions in China^6–8^, no investigation pertaining to the variations in root anatomy and mandibular first premolar has been conducted in the region of Guizhou, a southwestern province of China, which is a relatively closed region (with few immigrants from other regions) and a stable population.

The aim of this study is to investigate root anatomy and root canal morphology of human mandibular first premolars, as well as the correlation between root canal configuration and Tome’s root grading in a population from southwestern China.

## Results

### Number of Roots and Canals

Nearly all mandibular first premolars have a single root (177/178). Two roots were found in only 0.6% of the teeth studied (1/178). Of 178 mandibular first premolars, 64.04% possessed single-canal system, whilst 34.27% had two canals and 1.69% had three canals.

According to Vertucci’s classification, Type I canal system was found in 114 teeth (64.04%), Types II, III, IV, V and VIII in 2 teeth (1.12%), 19 teeth (10.67%), 1 tooth (0.56%), 39 teeth (21.91%), and 2 teeth (1.12%) respectively. And only one tooth (0.56%) belonged to an additional type (1-3-1)(Table 1).

**Table 1:** Distributions and percentages of categories of variants in the root canal anatomy of mandibular first premolars according to Vertucci’s criteria^21^.

	Type I 1	Type II 2-1	Type III 1-2-1	Type IV 2	Type V 1-2	Type VIII 1-3	Other 1-3-1	C-shaped (cross-section)
Number total: 178	114	2	19	1	39	2	1	22
Percentage	64.04%	1.12%	10.67%	0.56%	21.91%	1.12%	0.56%	12.36%

### Apical Foramina, Apical Deltas and Lateral canals

In the apical region, single apical foramen was found in 76.4% of the teeth studied. The incidence of two or more foramina is low at approximately 23.6%. The apical foramina in nearly half of sample teeth (46.63%) were located laterally. Of all teeth studied, apical deltas were found in 10.11% of teeth. Lateral canals were frequently seen (39.89%). The position of apical foramina and the location of the lateral canals and intercanal communications are shown in Table 2.

**Table 2:** Number of teeth with lateral canal, Intercanal communication or apical deltas and location of apical foramen, lateral canal or intercanal communication.

	Position of AF		Number of teeth with LC	Location of LC*			Number of teeth with ICC	Location of ICC*			Apical deltas
	central	lateral		Coronal	Middle	Apical		Coronal	Middle	Apical	
Number (178)	95	83	71	5	39	40	19	2	12	6	18
Percentage	53.37	46.63	39.89	5.95	46.43	47.62	10.67	10	60	30	10.11

AF: apical foramen; LC: lateral canal; ICC: Intercanal communication.

*Some samples have more than one lateral canals or intercanal communications.

### Radicular Grooves

**Figure 1:**
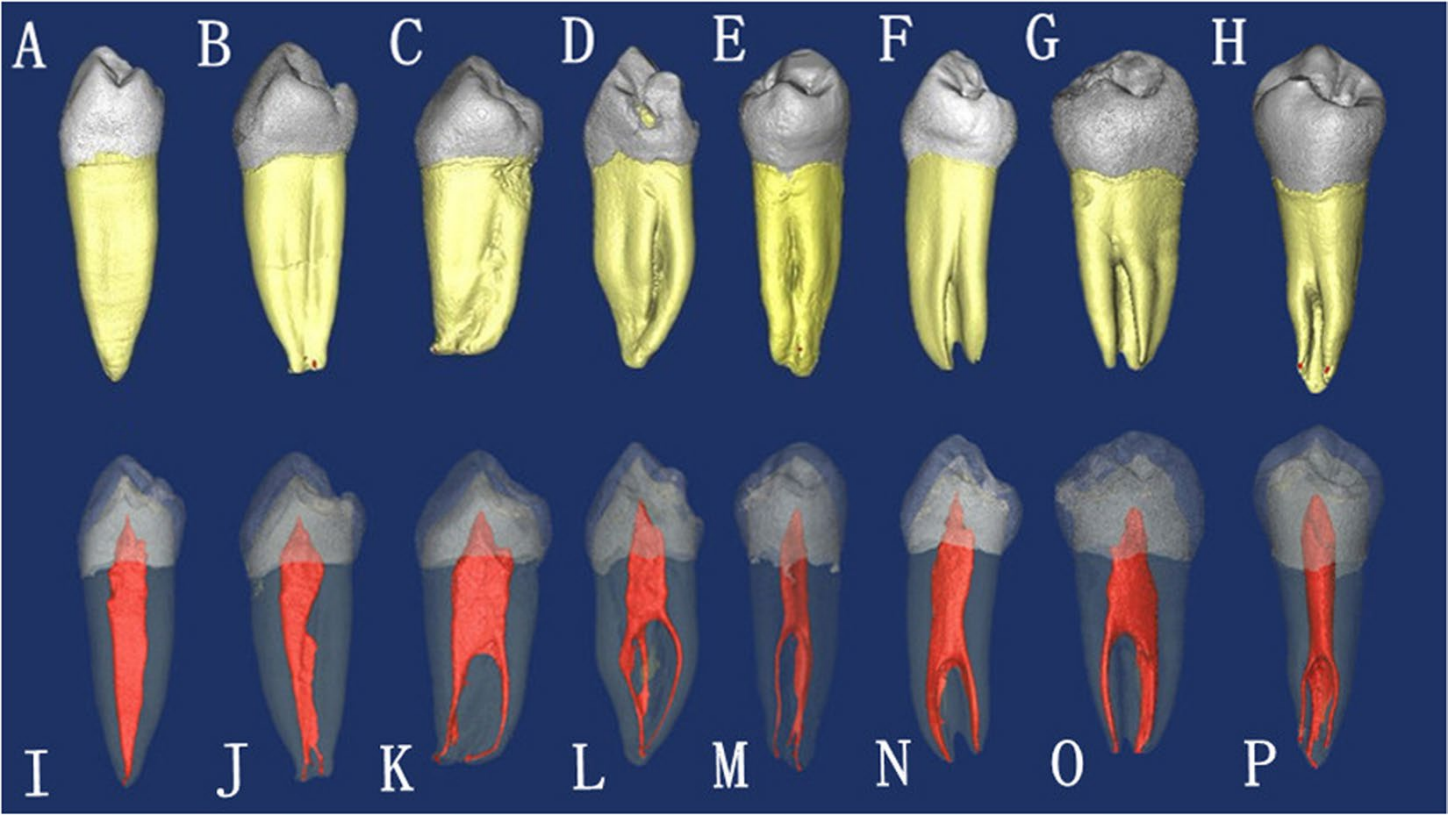
The micro-CT images showing external root anatomy and corresponding inner root canal morphology. The teeth were scored for radicular grooves variations according to ASUDAS. (**A**) grade 0, (**B,C**) grade 1, (**D**) grade 2, (**E,F**) grade 3, (**G,H**) grade 4, (**I–P**) the corresponding inner root canal morphology for teeth in (**A**–**H**).

Of the 178 mandibular first premolars studied, 44.38% had radicular grooves. Most of the grooves were located on the mesial surface of the root, followed by the lingual surface and distal surface in all teeth with radicular grooves. In 1.69% of samples the groove extended on the lingual and one proximal root sides. And 2.81% of samples had radicular grooves on both mesial and distal root surfaces. Only 1.12% of samples had radicuar grooves on all mesial, distal and lingual surfaces (Fig. 1). The distribution of the radicular grooves is shown in Table 3.

**Table 3:** The scoring on radicular grooves of mandibular first premolars studied according to Arizona State University dental anthropology scoring system.

	grade 0	grade 1	grade 2	grade 3	grade 4	grade 5^*^
Number of teeth	101	30	22	18	6	1
Percentage of two-canal system	0	70	86.36	87.5		0
Percentage of three-canal system	0	0	0	12.5		0
Percentage of C-shaped configuration	0	6.67	18.18	66.67		0

^*^The tooth (AUS = 5) has two root, each root has only single canal.

**Figure 2:**
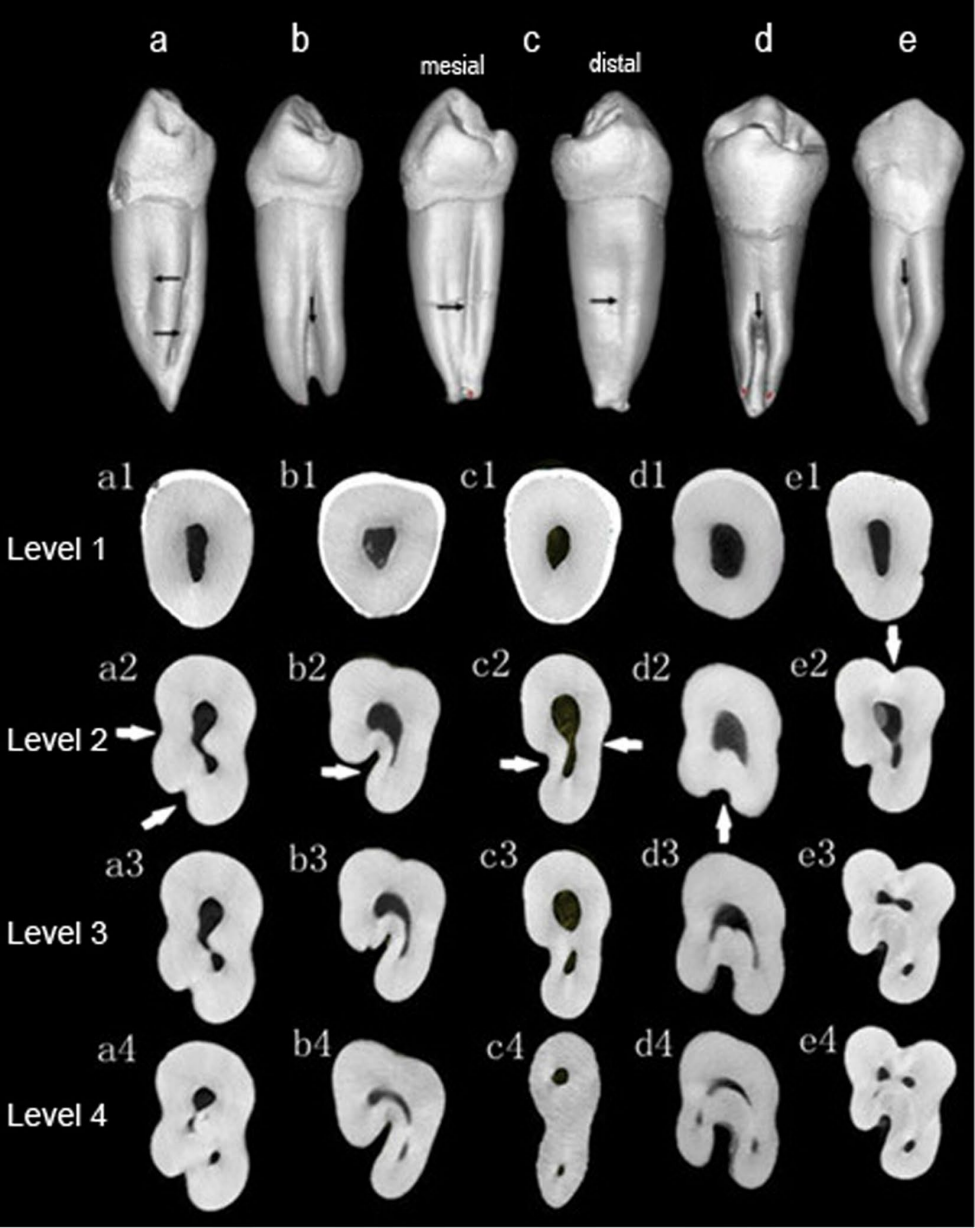
The micro-CT images showing the radicular grooves and cross-sectional configuration. Radicular grooves are shown on the external surfaces from proximal view (black arrow) and cross-sectional view (white arrow). From cross-sectional view, C-shaped configuration can be found in these samples. Level 1, cementoenamel junction; Level 2, coronal third; Level 3, middle third; Level 4, apical third.

According to ASUDAS, the ratings of radicualr grooves in samples were 56.74%(101/178), 16.85%(30/178), 12.36%(22/178), 10.11%(18/178), 3.37%(6/178) and 0.56%(1/178), respectively from grade 0 to grade 5 (Table 4). 24 sample teeth (13.48%) meet the criteria of Tome’s anomalous root (grade 3 or 4). All teeth with Tome’s anomalous root had two or more canals system, and 66.67% of them possessed C-shape configurations from the cross-sectional view (Fig. 2). In contrast, of all teeth studied, only 12.36% had C-shape configurations. Scores for radicular grooves are found to have a significant correlation with incidence of multiple canal (P < 0.001) and C-shape configurations (P < 0.001).

**Table 4:** The distribution of the radicular grooves in mandibular first premolars.

	Mesial surface	Lingual surface	Distal surface	Buccal surface	Both lingual and one proximal surfaces	Lingual and both proximal surfaces
Number (of teeth)	74	5	5	2	3	2
Percentage	41.57	2.81	2.81	1.12	1.69	1.12

## Discussion

The current study shows 35.96% of mandibular first premolars have multiple-canal system, which is consistent with the previous study^9^ on Chinese populations. However, the actual rate of detecting two canals is relatively low in clinical practice. It is inferred that the additional canal of permanent mandibular first premolars was often missed. The inability to locate, debride and obturate a second canal is often a major reason for failure when employing endodontic treatment for permanent mandibular first premolar. Consequently several measures should be adopted to reduce the chances of missing a canal. A good measure is the application of Cone-beam CT in a pre-treatment evaluation of root canal morphology. Despite its relative cost, and dosage difference that may hinder its use for individual patients, the cone-beam CT should be used as a first hand supplement when the roots seem irregular in 2D X-ray films^10^. A dental operating microscope should be used to help locate the possible additional root canal orifice during the endodontic treatment. The dentists should assume that an additional canal may exist and make a thorough search for a second canal. Inclusively it is paramount to poses a step-wise knowledge of where and how to search for a second and in some cases a third canal with endodontic treatment on a permanent mandibular first premolars. In several cases, the access to the buccal canals would be easy, but the lingual canals will prove otherwise. Adequate access cavities should be made to expose all orifices, and in some cases, the additional canal will be located only if the access cavity preparation is extended more lingually.

As earlier stated, root anatomy and root canal morphology usually vary in different ethnic groups^2, 10^. A previous study^11^ found that the incidence of multiple roots was much greater in the African American patients (16.2%) compared with white patients (5.5%) in the mandibular first premolar teeth. However, only 0.6% of sample teeth had two roots in this current study, which is close to that of an Indian population (2.9%)^12^. In respect of canals, Lu *et al*.^6^ reported a high incidence (46%) of multiple-canal system in Chinese population, while 62.61%, 39.5%, 28% and 19.4% were reported in Indian^12^, Turkish^13^, Jordanian^14^ and Japanese^15^ population respectively. There are also some variations in root anatomy and root canal morphology among people living in different regions of China. Sample teeth in this current study, collected from Guizhou province, have 35.96% incidence of two or more canals, which is similar to the study (34.8%) from Chongqing municipality^8^ in China. However, compared with the current study, a lower incidence were reported in populations from Shanghai municipality (16.5%)^16^ and Shandong province (22.86%)^7^.

The C-shaped canal system is an anatomical variation mostly seen in mandibular second molars. Although the incidence is low, C-shaped canal system can be also found in mandibular first premolars. It was reported that the incidence of C-shaped canals in mandibular first premolars is 10–18%^17^. The C-shaped canal variation of morphology occurs more frequently in Chinese people and can lead to difficulties during treatment in such population^18^. The main anatomic feature of C-shaped canals is the presence of a fin or web connecting the individual root canals. With irregular configuration, C-shaped canal cannot be well instrumented with nearly all types of NiTi rotary files. Hence biomechanical preparation combining NiTi instrumentation, ultrasonic irrigations and intracanal medications should be considered in order to guarantee a steriled environment prior to obturation. Besides, a newly-designed rotary file named XP-endo Finisher should be recommended and it is more effective to remove the debris and smear layer inirregular root canals^19^.

The radicular groove may be regarded as a developmental invagination, which can be found in the majority of mandibular first premolars. The presence of a groove, especially deep groove may increase the incidence of multiple-canal system. Moreover, the obvious groove results in the C-shaped root cross-section especially when Tome’s anomalous root exist, which may increase the difficulty of endodontic treatment. In the current study, a significant correlation between Tome’s anomalous root and complex root canal configurations has been demonstrated. According to previous Anthropological study^20^, Tome’s anomalous root is considered to be a normal morphologic variant and can be identified as a non-metric dental trait of Asian population. Therefore, recognizing the existence of the grooves, especially in an Asian population, provide a hint to the clinicians.

An additional reason for the difficulty of endodontic treatment of mandibular first premolars is the prevalence of apical deltas, inter-canal communications and lateral canals, so thorough knowledge and proper diagnosis of this situation is vital before treatment. Furthermore, biomechanical preparation and sealer with perfect flow-ability should be applied during the treatment. The apical foramen was located laterally in nearly half of the teeth in the current study. When the foramen opens laterally, the working length may appear to be short on the radiograph. In this condition, the working length is confirmed repeatedly by electronic apex locater but judged by radiograph.

Micro-CT is an important tool for the *in-vitro* evaluation of the root canal morphology because of its high resolution and non-destructive nature. With the 3D analysis software, it is convenient to view cross sections of sample teeth and correlate external anatomy with internal canal morphology.

There is obvious variation of the root anatomy and root canal morphology of mandibular first premolar among southwestern Chinese population, which is very complex and requires careful assessment for endodontic treatment.

## Methods

178 extracted human mandibular firstpremolars were collected from hospitals and dental clinics in Guizhou, asouthwestern province of China. A written informed consent was obtained from each donor. Approval for the study was obtained from the institutional ethical committee of Chongqing Medical University. **All methods were performed in accordance with the relevant guidelines and regulations**. Teeth included in this study had clinical crowns without major defects, complete root structures and fully developed apices. The teeth with defect/caries over 1/2, root canal fillings, crown restorations, open apices, or fracture were excluded.

All teeth were placed in 5.25% sodium hypochlorite for 1 hour, and then any remaining soft tissue or calculus were removed by scaling. For each sample, the tooth length from apex to cusp and the root length from apex to cemento-enamel junction were measured by a vernier caliper. Each sample was examined for the number of roots, the radicular grooves or depressions on all root surfaces using a stereomicroscope (M50, Leica, Germany).

**Figure 3:**
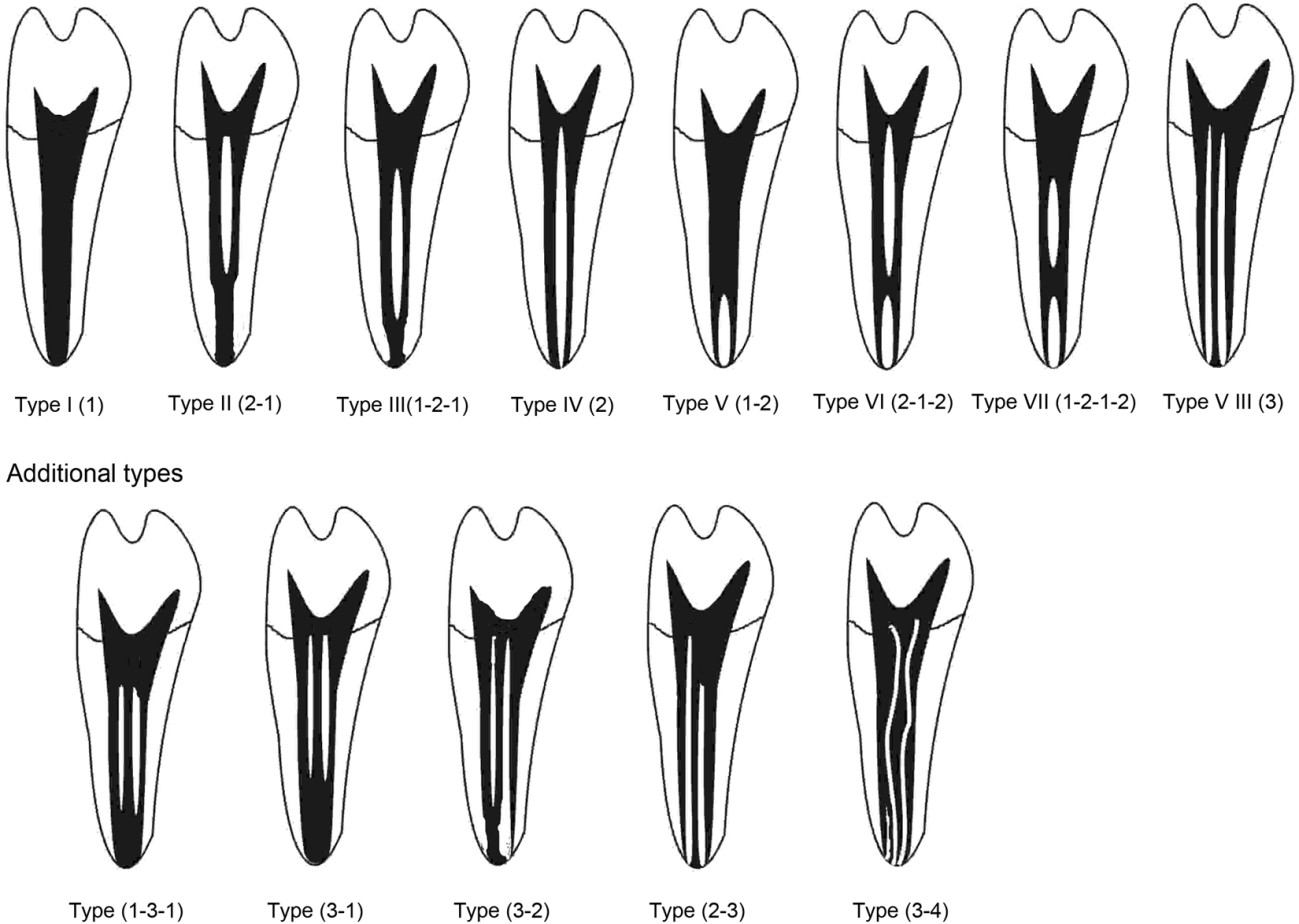
Illustration showing the categories of root canal morphologies in human permanent teeth according to the method by Vertucci^21^.

All samples were scanned using a micro-CT scanner (micro-CT Inveon; Siemens Medical Solutions, Knoxville, TN) with voxel sizes of 15 μm × 15 μm × 15 μm. The in-built Cobra software (Siemens Medical Solutions, Knoxville, TN) was used for the 3D reconstruction and analysis. The transparency of the model, viewing angle, and magnification were adjusted to display external and internal root structures clearly. The 3D images were investigated as below: (i) number and type of root canals; (ii) frequency and location of lateral canals and intercanal communications; (iii) location of apical foramina (central at root tip or lateral); and (iv) frequency of apical deltas. The root canal configuration was categorized and compared according to Vertucci’s criteria^21^ (Fig. 3). The external surface of teeth like radicular grooves or bifurcations was also examined. The radicular grooves were graded according to the Arizona State University dental anthropology scoring system (ASUDAS)^4^. The Tome’s anomalous root was defined when the score is equal to 3 or 4 (Table 5). All data were analyzed using the SPSS software (version 19.0). The correlation between scores for radicular grooves and root canal morphology was analyzed using Spearman rank correlation.

**Table 5:** Classification of Tome’s root according to Arizona State University dental anthropology scoring system^4^.

Score	Description	Tome’s anomalous root
Grade 0	RG is absent, or if present, shallow with rounded indentation	
Grade 1	RG is present and has a shallow V-shaped cross-section	
Grade 2	RG is present and has a moderately deep V-shaped cross-section	
Grade 3	RG is present, V-shaped, and deep. Groove extends at least 1/3 of total root length	Yes
Grade 4	RG is deeply invaginated on both the mesial and distal root surfaces	Yes
Grade 5	Two free roots are present. Their length is at least 1/4 to 1/3 of the total root length.	

RG: radicular groove.
